# Some Relationships of Secondary Parotitis

**Published:** 1905-08-26

**Authors:** 


### SOME RELATIONSHIPS OF SECONDARY PAROTITIS.

An interesting observation which bears on the question of the mode of causation of secondary or symptomatic parotitis is recorded by Messrs. Hastings and Hillier.[1] It has long been a matter of dispute whether in these cases the irritant reaches the gland by the blood-stream, or whether, on the other hand, infection takes place from the buccal cavity through Stenson's duct. The case now reported gives support to the latter, rather than to the former view. The patient was a man of 56 years who attended the out-patient department of the Middlesex Hospital for an inflammatory enlargement of the right parotid gland. Bacteriological examination of the saliva obtained from the gland gave a nearly pure culture of a diplococcus, whilst tubes inoculated with the secretion from the left cheek remained sterile. The unilateral character of the infection is, of course, suggestive of a local origin, and the condition of the teeth and buccal cavity generally was one in which duct infection might readily occur. Further, whilst the left salivary papilla presented a normal appearance, the right papilla was reddened, bled readily when touched, and was unduly prominent.

Parotitis in the course of lobar pneumonia has been observed by several authors, and there is much difference of opinion both as to its gravity and also as to its exact relationship to the primary disease. Grisolle,[2] who has fully discussed the subject, regards parotitis as a very serious complication of pneumonia, the cases, in his experience, usually terminating in suppuration or gangrene, and the effect on the patient's prospects of recovery being decidedly prejudicial. This gloomy view, however, has not met with general acceptance, and instances have been quoted in which the gland did not suppurate and in which also the parotitis seemed in no way to retard a favourable issue. On the whole it would appear that when parotitis occurs during the height of the disease it is a more serious fact than when it develops after the occurrence of the crisis. Quite a number of cases have been observed in the latter circumstances and in most of them resolution has been successfully accomplished. It may be added that examination in some of these instances of parotitis in pneumonia has revealed the presence of the pneumococcus both in the blood and also in the parotid fluid. Altogether it must be said that the study of parotitis as a complication of pneumonia still leaves the question of the route of infection an unsolved problem.

A case of parotitis, secondary to a gonococcal urethritis, is reported by Dr. Chas. A. Powers.[3] Incisions had been made for a swelling of the right elbow region, and a quantity of thin pus had been evacuated. Subsequently there were suggestions of a general gonococcal infection, and the left parotid gland was painful and tender. In the end, however, the alarming features of the case subsided, and the patient made a good recovery except for some permanent stiffness of the right elbow and wrist joints. In this instance the swelling of the parotid gland appeared to be part of a generalised septic process rather than an example of the parotitis which, as an isolated phenomenon, is known to occur in various febrile conditions and after operative procedures involving the abdominal and pelvic viscera. A good example of this latter variety is reported by Mr. F. B. Jefferies,[4] after an operation for the relief of a strangulated hernia. The patient was a woman of 68 years in whom, three days after the operation, the right parotid gland showed evidences of severe inflammation, and the temperature rose to 103°. There were no evidences of peritonitis and no other local inflammatory developments suggestive of a generalised sepsis. The parotitis caused much distress and interfered seriously with deglutition. Death occurred about 24 hours after its onset.

An observation that bears upon the etiology of secondary parotitis is the development of pancreatitis in "mumps." In that disease, as is well known, complications in the form of secondary inflammations, such as orchitis and mastitis, are possible, and in view of the similarity in structure of the glands involved it is hardly a matter for surprise to learn that pancreatitis may sometimes complicate parotitis. Hitherto this has lacked the evidence of positive demonstration, but in an instance recently reported[5] the position was made certain by post-mortem examination. The patient, a man of 19 years, was admitted to hospital suffering from "mumps," the disease running a favourable course with restoration of normal temperatures on the fifth day. On the tenth day there was a rigor followed by fever and swelling of the right testicle. In the course of a few days, however, the orchitis subsided and the patient again appeared convalescent. But on the fifteenth day he was found to be somewhat prostrate, though the temperature was normal and the pulse slow. He complained of a sense of weight in the epigastrium and had vomited two or three times. There was tenderness in the epigastrium and over the region of the gall-bladder, the spleen was enlarged and the conjunctivæ had an icteric tint. Later, jaundice became more distinct and vomiting was extremely troublesome. Ultimately hæmatemesis occurred, and the patient became unconscious and died. At the autopsy the pancreas was found to be greatly enlarged, œdematous and congested; and there was much swelling of all the lymphatic glands in its neighbourhood. Microscopical examination showed enlargement of the acini of the gland with compression of the islands of Langerhans by the turgescent tubes; the nuclei of the pancreatic cells stained badly and many of them were vesicular. The liver, spleen, and kidneys showed nodules of embryonic cells such as are common to all infectious processes.

[1] Lancet, Aug. 12, 1905. [2] Ziemssen's Cyclopædia. [3] Med. Rec., Oct. 3, 1903. [4] Lancet, Dec. 12, 1903. [5] Ibid, Aug. 12, 1905.

